# Thrombophilia Testing After VTE and in Related Clinical Contexts: Is It Necessary, and Does It Change Management? Appraisal of Evidence, Guidelines, and Clinical Yield

**DOI:** 10.3390/jcm15197393

**Published:** 2026-09-23

**Authors:** Riwad Iftekhar, Emory Ware, Emilee Ly, Mohammad I. Ullah

**Affiliations:** 1 School of Medicine, University of Mississippi Medical Center, Jackson, MS 39216, USA; 2 Department of Internal Medicine, University of Mississippi Medical Center, Jackson, MS 39216, USA; mullah@umc.edu

**Keywords:** thrombophilia, venous thromboembolism, antiphospholipid syndrome, anticoagulation, Factor V Leiden, thrombophilia testing, clinical practice guidelines

## Abstract

Thrombophilia testing is commonly performed after venous thromboembolism (VTE), yet its clinical value is frequently overestimated. This narrative critical review synthesizes key observational studies, systematic reviews, and contemporary clinical practice guidelines addressing the utility, timing, and management implications of thrombophilia testing after VTE, as well as in related clinical settings where analogous testing decisions arise such as cryptogenic arterial stroke and recurrent pregnancy loss. Evidence and recommendations were selected based on their clinical relevance to anticoagulation decisions, recurrence risk assessment, and patient-specific management, with particular attention to contemporary guidelines from the American Society of Hematology, American College of Chest Physicians, National Institute for Health and Care Excellence, and British Society for Haematology. The strength and certainty of the evidence underlying these recommendations were also considered, recognizing that several recommendations are conditional and are supported by very low certainty evidence and decision modeling. Overall, the available evidence synthesized in this review indicates that anticoagulation decisions are determined primarily by whether the event was provoked or unprovoked, the risks of recurrence and bleeding, and patient-specific clinical factors rather than by the presence of most inherited thrombophilias. Although thrombophilia testing often identifies laboratory abnormalities, it rarely changes clinical management while increasing healthcare costs and the potential for unnecessary treatment and patient anxiety. Antiphospholipid syndrome remains the major exception because its diagnosis directly influences anticoagulant selection, treatment duration, and pregnancy management. This review also considers the importance of appropriate test timing, the effects of anticoagulant therapy on test interpretation, and the role of thrombophilia testing in selected clinical scenarios. Based on the available evidence, we propose a practical, management-oriented approach in which testing is reserved for situations where the results are expected to influence therapeutic decision making, thereby promoting high-value, evidence-based patient care.

## 1. Introduction

Despite decades of expanding thrombophilia panels and increasing test utilization, the clinical utility of thrombophilia testing after venous thromboembolism (VTE) remains uncertain. Venous thromboembolism, encompassing deep vein thrombosis and pulmonary embolism, remains a major cause of preventable morbidity and mortality worldwide. Following a thrombotic event, clinicians and patients are often driven to identify an underlying cause, and thrombophilia testing has become a reflexive component of that evaluation. The premise is intuitive: identification of an underlying hypercoagulable state may explain the event, refine recurrence risk assessment, and guide treatment duration. In practice, however, the question of whether to test is substantially more complicated.

For most patients, thrombophilia testing results do not meaningfully alter management. Decisions regarding anticoagulation duration are driven primarily by whether thrombosis was provoked or unprovoked, recurrence risk, bleeding risk, and patient-specific clinical factors rather than by inherited thrombophilic genotype alone [1]. Although inherited thrombophilias increase relative thrombotic risk, recurrence after VTE is influenced more strongly by provoking factors and overall clinical context. Nevertheless, thrombophilia testing continues to be performed routinely in many institutions despite guideline recommendations favoring selective and clinically targeted use [1,2]. Positive results are frequently overinterpreted, resulting in prolonged anticoagulation in patients with otherwise low recurrence risk, while negative results may generate false reassurance.

The economic implications of indiscriminate testing are also substantial. Institutional audits have demonstrated that thrombophilia testing influences clinical management in fewer than 20% of tested patients despite significant associated healthcare expenditures [2,3]. Importantly, the harm of over testing extends beyond financial cost alone. False-positive or clinically insignificant results may expose patients to unnecessary anxiety, additional testing, prolonged anticoagulation, and the burden of a lifelong diagnostic label.

Despite these limitations, thrombophilia testing cannot be dismissed entirely. Certain clinical scenarios remain highly actionable, particularly antiphospholipid syndrome (APS), where diagnosis directly influences anticoagulant selection, duration of therapy, pregnancy management, and arterial thrombosis prevention [1]. The challenge therefore lies not in whether thrombophilia exists as a clinically relevant entity, but in identifying when testing meaningfully changes management.

This narrative review examines the evidence surrounding thrombophilia testing following VTE and related clinical settings. Literature was identified through targeted searches of PubMed/MEDLINE. Search terms included related to thrombophilia testing, VTE, recurrent risk, anticoagulation, antiphospholipid syndrome, and relevant society guidelines. The primary search period extended from January 2010 through June 2026, thereby prioritizing contemporary evidence while encompassing the period during which substantial advances occurred in the diagnosis, pathophysiological understanding, and management of VTE. Older foundational references were retained when directly relevant to contemporary understanding. Guideline recommendations identified key clinical questions and areas of uncertainty, guiding iterative searches for relevant observational studies, systematic reviews, meta-analyses, and other peer reviewed literature. The review discusses the clinical and economic considerations surrounding thrombophilia testing, compares contemporary guideline recommendations, examines selected clinical scenarios, and summarizes considerations informing practical, management-oriented approaches.

## 2. Pathophysiology and Classification of Thrombophilia

Thrombophilia refers to a predisposition toward thrombosis resulting from disruption of normal hemostatic balance [4]. Thrombophilic states may be inherited or acquired and involve abnormalities affecting coagulation factors, endogenous anticoagulants, endothelial activation, or fibrinolytic pathways.

Testable inherited thrombophilias include Factor V Leiden (FVL), prothrombin G20210A mutation, antithrombin deficiency, Protein C deficiency, and Protein S deficiency. Testable acquired thrombophilia is most prominently represented by antiphospholipid syndrome (APS), myeloproliferative neoplasms (JAK2, CALR, MPL mutations), and paroxysmal nocturnal hemoglobinuria. In contrast, acquired clinical risk states such as malignancy, pregnancy, inflammatory disorders, estrogen exposure, and recent surgery substantially elevate thrombotic risk but are not detected by conventional thrombophilia panels and require clinical recognition rather than laboratory confirmation [5].

The coagulation cascade is normally regulated by endogenous anticoagulant systems including antithrombin and the activated protein C pathway. In physiologic coagulation, factor Xa and factor Va form the prothrombinase complex, which converts prothrombin to thrombin; thrombin then promotes fibrin formation and further amplifies coagulation (Figure 1). This process is normally limited by natural anticoagulants such as antithrombin and the protein C system in which activated protein C with the cofactor Protein S inactivates factors Va and VIIIa. Dysfunction in any step of the coagulation pathway results in various inherited and acquired thrombophilias.

In FVL, mutation of the activated protein C cleavage site renders factor V resistant to degradation, resulting in persistent procoagulant activity [6]. In prothrombin G20210A mutation, elevated prothrombin levels increase thrombin generation and fibrin formation [4]. APS represents a distinct acquired autoimmune thrombophilia characterized by antiphospholipid antibodies that activate endothelial cells, platelets, complement pathways, and tissue factor–expressing monocytes [7].

Importantly, thrombophilia status alone is an imperfect predictor of thrombosis. Many individuals with inherited thrombophilia remain asymptomatic throughout life, and absolute thrombotic risk varies substantially according to provoking factors such as surgery, immobilization, inflammation, pregnancy, and estrogen exposure [8,9]. These observations reinforce the principle that thrombophilia testing results must be interpreted within broader clinical context rather than in isolation.

## 3. Prevalence of Common Thrombophilia Conditions

While thrombophilia as a category encompasses a wide spectrum of inherited and acquired disorders, these conditions differ substantially in their prevalence in the general population and in the magnitude of thrombotic risk they confer. Understanding prevalence data is essential for contextualizing testing decisions: high population prevalence does not imply high individual risk, and low prevalence does not preclude clinical significance. The following section summarizes the epidemiology of the most commonly encountered thrombophilias, drawing on cohort studies, family-based prospective data, and meta-analyses.

Of note, the methylenetetrahydrofolate reductase (MTHFR) C677T polymorphism is among the most common genetic variants included in thrombophilia panels, with homozygosity occurring in approximately 10–15% of the general population and heterozygosity in 30–40%. Despite this high prevalence, MTHFR homozygosity is not independently associated with increased VTE risk when folate and vitamin B12 levels are normal, and it is not associated with a clinically meaningful increase in thrombotic risk in individuals with adequate nutritional status [1]. For this reason, MTHFR C677T polymorphism should not be included in thrombophilia classifications or included in panel testing. Contemporary guidelines explicitly discourage MTHFR testing in the evaluation of patients with VTE. However, it continues to appear on many commercial thrombophilia panels and consistently generates a positive result when testing for thrombophilia without producing management changes [1,2].

It is important to remember that incidence estimates of VTE in asymptomatic carriers are derived predominantly from prospective and retrospective family cohort studies of relatives of thrombotic probands. These populations may have enriched genetic backgrounds or shared environmental factors that differ from unselected carriers in the general population, and therefore incidence rates reported in family studies may not be directly generalizable to all carriers. Additionally, ascertainment of family members may preferentially include relatives with subclinical thrombotic events, potentially overestimating true population-level event rates. With these limitations in mind, the following family cohort derived estimates provide useful context for clinical decision-making.

### 3.1. Factor V Leiden

Factor V Leiden (FVL) is the most common heritable thrombophilia identified in populations of European descent. The heterozygous mutation has an estimated prevalence of 3–8% in the White European and European-American population, approximately 2% in Hispanic Americans, 1% in African Americans, and 0.5% in Asian Americans, reflecting a marked ethnic variation in carrier frequency [10,11].

Despite its prevalence, the absolute annual risk of VTE in asymptomatic heterozygous FVL carriers is modest. In the retrospective family cohort by Simioni et al., annual total and spontaneous VTE incidences among FVL carriers were 0.28% and 0.11% respectively, compared with 0.09% and 0.04% among non-carriers, yielding relative risks of 2.8 (95% CI 1.1–8.6) and 2.5 (95% CI 0.6–10.6) [9]. Additional provoking factors such as oral contraceptive use and pregnancy increased the thrombotic risk in FVL carriers to 3.3-fold and 4.2-fold, respectively. These data collectively indicate that heterozygous FVL confers a moderate relative risk elevation but a low absolute annual risk in asymptomatic individuals, which limits the clinical utility of routine screening [8,9]. However, homozygous FVL is rare and represents a qualitatively different risk category: it is associated with substantially greater thrombotic risk with a primary VTE OR of 7.38 (95% CI 4.68–11.65) compared to noncarriers and is classified as a high-risk thrombophilia [12].

### 3.2. Prothrombin G20210A Variant

The prothrombin G20210A variant is the second most common inherited thrombophilia in European-descent populations, with a heterozygous prevalence of approximately 1–3% [13,14]. The mutation leads to elevated prothrombin levels, promoting thrombin accumulation and fibrin clot formation [4]. Compared with the general population, patients with this mutation have a 3-fold increased risk of VTE [15]. Annual VTE incidence in asymptomatic heterozygous carriers is estimated at approximately 0.4% (95% CI 0.1–1.1) per year from family cohort data, making the absolute risk low in the absence of additional triggers [16].

As with FVL, compound heterozygosity (FVL plus prothrombin G20210A) or double heterozygosity markedly amplifies thrombotic risk and is generally classified in the high-risk category. The isolated prothrombin G20210A variant, while a significant risk modifier, carries a lower absolute thrombotic risk than deficiencies of natural anticoagulants [12].

### 3.3. Antithrombin, Protein C, and Protein S Deficiencies

Deficiencies of the natural anticoagulants including antithrombin (AT), Protein C (PC), and Protein S (PS) are considerably less common than FVL or the prothrombin variant but are associated with substantially higher thrombotic risk. Estimates of general population prevalence vary by method and population but are approximately 0.02–0.04% for AT deficiency, 0.2–0.4% for PC deficiency, and 0.03–0.13% for PS deficiency [17,18].

In the landmark retrospective cohort study by Simioni et al., annual total and spontaneous VTE incidences among carriers of AT, PC, or PS deficiencies were 1.01% and 0.40%, respectively, compared with 0.10% and 0.04% in non-carriers with a relative risk of 10.6-fold for both outcomes [9]. The prospective cohort study by Sanson et al. enrolled 208 asymptomatic family members of affected probands and reported an overall annual VTE incidence of 1.5% (95% CI 0.7–2.8) across the three deficiencies combined, with antithrombin deficiency carrying the highest individual risk at 1.6% per year, followed by protein C deficiency at 1.0% and Protein S deficiency at 0.4% [19]. Additional risk factors, including surgery, immobilization, oral contraceptive use, and pregnancy, further amplified VTE risk in carriers by 6- to 8-fold compared with non-carriers [9].

A subsequent prospective family cohort study by Tormene et al. reported an annual VTE incidence of 0.55% among 139 carriers of clotting inhibitor defects without the HR2 factor V haplotype which was somewhat lower than in earlier retrospective studies, likely reflecting selection differences [20]. The multicenter European family study by Bucciarelli et al. reported an overall VTE incidence of 0.52 per 100 patient-years across AT, PC, and PS deficiency groups combined, with antithrombin deficiency again carrying the highest individual risk [18]. A 2024 systematic review and meta-analysis by Alnor et al. confirmed that while relative risks remain significantly elevated for all three deficiencies, absolute event rates are somewhat lower than previously estimated in older retrospective studies, underscoring the importance of basing risk estimates on prospective data [12].

Collectively, these studies confirm that AT, PC, and PS deficiencies carry a higher absolute annual risk of VTE than FVL or the prothrombin variant and are appropriately classified as high-risk thrombophilias. Nevertheless, the absolute annual event rates remain sufficiently low, typically well below 2% per year in asymptomatic carriers so that indefinite anticoagulation is not warranted without additional clinical context [9,19].

### 3.4. Antiphospholipid Syndrome

Antiphospholipid syndrome (APS) differs fundamentally from the inherited thrombophilias in that it is an acquired autoimmune disorder. The prevalence of antiphospholipid antibodies (aPL) including lupus anticoagulant, anticardiolipin antibodies, and anti-β2-glycoprotein I antibodies in the general adult population ranges from approximately 1–5% when broadly defined by seropositivity alone. However, the prevalence of clinically diagnosed APS, requiring both aPL positivity and a thrombotic or obstetric clinical event meeting Sapporo/Miyakis criteria, is considerably lower, estimated at approximately 0.05% of the general population but 1–5% of individuals presenting with VTE [21,22]. While the 2006 revised Sapporo criteria historically defined APS classification, the 2023 ACR/EULAR criteria provide an updated, weighted classification system designed for research cohort homogeneity. In clinical practice, APS diagnosis remains multifactorial and based on individualized clinical assessment rather than strict algorithmic application of clinical criteria.

The risk conferred by APS depends heavily on antibody profile. Triple-positive APS, defined as concurrent positivity for lupus anticoagulant, anticardiolipin antibodies, and anti-B2-glycoprotein I antibodies, confers the highest thrombotic risk. In patients with a prior thrombotic event and triple-positive antibodies receiving inadequate anticoagulation, annual thrombotic recurrence risk has been estimated to approach or exceed 29%. With appropriate vitamin K antagonist therapy (target INR 2.0–3.0 for VTE), the risk is reduced, though specific recurrent rates with adequate anticoagulation vary by clinical context. Conversely, asymptomatic aPL-positive individuals without prior thrombotic events should receive individualized risk assessment based on aPL antibody profile, clinical context, and other thrombotic risk factors [22]. A major unresolved question in APS pathophysiology is why thrombosis occurs as discrete clinical events despite the continuous presence of circulating aPL. The prevailing explanation is the “two-hit” model: aPL constitute a persistent prothrombotic first hit that primes endothelial cells, platelets, monocytes, and complement, but a second triggering event such as infection, surgery, immobilization, pregnancy, or other inflammatory stimuli is typically required for frank thrombosis to develop [23,24].

The clinical importance of APS within the thrombophilia landscape is particularly important. As an acquired disorder, it can present at any age and is not predicted by family history. Moreover, its management implications are distinct from those of inherited thrombophilias. A confirmed APS diagnosis may necessitate vitamin K antagonist therapy rather than a direct oral anticoagulant, and it has implications for anticoagulation duration which substantially changes obstetric monitoring and prophylaxis plans [7]. For all these reasons, APS testing commands a uniquely prominent position across all major guideline recommendations. Table 1 summarizes the epidemiological data and risk stratification detailed above.

Taken together, these prevalence data highlight a fundamental epidemiological paradox in thrombophilia: the most common inherited variants (FVL, prothrombin G20210A) carry a relatively modest absolute annual risk that rarely justifies treatment escalation on its own, while the rarest deficiencies (antithrombin, Protein C, and Protein S) carry the highest individual risk but are so infrequent that their overall contribution to VTE burden in the population is limited. Antiphospholipid syndrome occupies a distinct position as an acquired thrombophilia whose prevalence, risk profile, and management implications are each qualitatively different from the heritable variants. These distinctions underpin the risk-stratified and management-driven approach to thrombophilia testing advocated across contemporary guideline frameworks, and they explain why a positive result alone without a specific clinical question rarely constitutes sufficient justification for testing.

## 4. Current Guideline Landscape

The clinical question of when to test for thrombophilia has been addressed by multiple major guideline-producing bodies across North America, the United Kingdom, and Europe. The four sources producing the recommendations include the American Society of Hematology (ASH) 2023 thrombophilia testing guidelines, the American College of Chest Physicians (CHEST) 2021 s update on antithrombotic therapy for VTE, the National Institute of Health and Care Excellence (NICE) guideline NG158 on venous thromboembolic diseases, and the British Society for Hematology (BSH) thrombophilia testing guideline. All major guidelines discourage routine thrombophilia testing after clearly provoked VTE, particularly when anticoagulation decisions are already determined by provoking factors. In addition, antiphospholipid syndrome (APS) occupies a uniquely important role across all frameworks because diagnosis frequently alters anticoagulant selection and duration of therapy.

ASH 2023 provides the most comprehensive and risk-stratified thrombophilia testing framework [25]. The guideline conditionally recommends based on low certainty against testing in unprovoked VTE because patients would likely initiate indefinite antithrombotic therapy regardless of test results. Similarly for surgically provoked VTE, patients would likely discontinue anticoagulation after completion of primary treatment regardless of results, which is why the guideline conditionally recommends against testing based on low certainty of evidence. However, ASH supports selective testing in hormonally associated VTE, nonsurgical provoked VTE, cerebral venous sinus thrombosis, and splanchnic vein thrombosis when anticoagulation duration remains uncertain. Selective testing in the situations listed above, however, assume indefinite anticoagulation, with the idea of eventual testing to determine continuation or cessation of anticoagulation. Overall, the ASH guidelines did not include direct comparative studies and were instead based on low certainty, indirect evidence and modeling.

CHEST 2021 does not function primarily as a thrombophilia testing guideline but indirectly shapes testing utility through its anticoagulation framework [26]. The guideline recommends against offering extended-phase anticoagulation in the setting of a major transient risk factor (surgery, immobility, major trauma) with a strong recommendation and moderate certainty of evidence. In the setting of a minor transient risk factor (estrogen, pregnancy, puerperium, reduced mobility), they also advise against extended anticoagulation with a weak recommendation and moderate certainty of evidence. If a patient has VTE in the absence of transient provocation (unprovoked or provoked by persistent risk factor), they strongly recommend extend-phase anticoagulation with a DOAC, based on moderate certainty evidence. If the patient cannot receive a DOAC, then they weakly recommend offering extended-phase anticoagulation with a VKA, based on moderate certainty evidence. If a patient is diagnosed with VTE in the setting of APS, they weakly recommend treatment with VKA over DOAC therapy with low certainty evidence. The guideline emphasizes provoking factors and recurrence risk rather than inherited thrombophilia status when determining anticoagulation duration. Under this framework, thrombophilia testing rarely changes management except in selected intermediate-risk situations and APS.

NICE NG158 adopts a comparatively restrictive approach. The guideline recommends against hereditary thrombophilia testing in patients continuing anticoagulation therapy. It also recommends against any type of thrombophilia testing in patients with provoked VTE, but the guideline does not stratify levels of provocation. For unprovoked VTE, NICE supports APS testing when the patient is planning to stop anticoagulation treatment and endorses hereditary thrombophilia evaluation if stopping anticoagulation and the patient has a first degree relative with PE or DVT. The guideline advises that anticoagulants may affect these results. These recommendations are not specified by a strength or certainty of evidence [27].

The BSH 2022 guideline also emphasizes restraint when testing for thrombophilia. The guideline begins by citing criteria for an inherited monogenic panel (AT, PC, or PS Deficiency), stating that testing should be reserved for patients younger than 40 with weak or no provocation and presence in a first degree relative. As a recommendation, BSH only endorses testing when results will change management (Grade 2B). For APS, BSH recommends testing for unprovoked or minorly unprovoked VTE since choice of antithrombotic therapy would be affected (Grade 1B). The BSH never makes an explicit recommendation about majorly provoked VTE; however, BSH overall discourages indiscriminate testing and emphasizes that evaluation should only proceed when clinically actionable [28].

Ultimately, these guidelines differ less in principle than in how they interpret uncertainty and define clinically meaningful actionability. All major frameworks support a transition away from reflexive thrombophilia panels toward targeted, management-directed testing. A comparison of the guidelines for certain clinical scenarios is provided in Table 2.

## 5. Timing of Thrombophilia Testing: When Should Testing Be Performed?

The timing of thrombophilia testing is a critical determinant of test accuracy and clinical usefulness. Testing performed during acute thrombosis or while a patient is receiving anticoagulant therapy may produce misleading results that can lead to misdiagnosis, unnecessary repeat testing, inappropriate anticoagulation decisions, and increased healthcare costs [1,29]. Consequently, major professional societies, including the American Society of Hematology (ASH), British Society for Haematology (BSH), and International Society on Thrombosis and Haemostasis (ISTH), recommend delaying most thrombophilia testing until the acute thrombotic episode has resolved and anticoagulation-related assay interference has been minimized [25,28].

### 5.1. Effect of Acute Thrombosis on Test Results

Acute thrombosis itself can alter several naturally occurring anticoagulant proteins. Protein C, Protein S, and antithrombin levels may decrease transiently during an acute thrombotic event due to consumption and inflammatory responses. Testing during this period may therefore falsely suggest hereditary deficiency and lead to inappropriate labeling of patients as thrombophilic [1].

Testing for APS is recommended shortly after the clinical event. However, acute phase reactants can alter the clotting-based assay of the lupus anticoagulant, and acute thrombosis can cause the appearance of transient anti-cardiolipin antibodies, affecting the solid-phase test. This nuance underscores the importance of confirmatory testing at least 12 weeks afterwards [7].

Genetic tests such as Factor V Leiden and Prothrombin G20210A variant analysis are not affected by acute thrombosis and may technically be performed at any time. However, because these results rarely influence immediate management, it’s not clinically necessary to order these tests during the initial presentation or hospitalization [1].

### 5.2. Effect of Anticoagulant Therapy on Testing

Anticoagulants may substantially interfere with thrombophilia assays, and the degree of interference depends on both the anticoagulant and the specific assay methodology used. Warfarin reduces Protein C and Protein S levels, potentially producing falsely low results that mimic hereditary deficiency. Testing should generally be deferred until warfarin has been discontinued for at least two weeks [28,29]. Unfractionated heparin and low-molecular-weight heparin may affect antithrombin measurements and lupus anticoagulant testing, particularly at therapeutic doses [29]. Direct oral anticoagulants (DOACs) may interfere with numerous clot-based assays, including lupus anticoagulant testing, activated protein C resistance assays, and antithrombin measurements. Depending on the specific assay, interference may produce both false positive and false negative results [30]. Chromogenic assays for Protein C and antigenic assays for Free Protein S are generally resistant to this interference and may be more reliable in patients on DOAC therapy, but Antithrombin chromogenic assay can be affected by DOACs [28]. Furthermore, when thrombophilia testing is being considered in a patient receiving a DOAC, consultation with the coagulation laboratory or hematology service may be appropriate to determine the potential assay interference, optimal timing, and methodology of testing.

### 5.3. Optimal Timing for Testing

For most patients, thrombophilia testing should be deferred until the acute thrombotic event and factors that may interfere with assay interpretation have been adequately addressed. The appropriate timing depends on the specific thrombophilia assay, anticoagulation exposure, and clinical question. Specific recommendations for individual tests are provided in Table 3.

### 5.4. Repeat Testing and Confirmation

A diagnosis of antiphospholipid syndrome requires persistence of at least one of the antiphospholipid antibodies (lupus anticoagulant, anticardiolipin antibody, or anti-β2-glycoprotein I antibody), on repeat testing at least 12 weeks apart, in addition to an appropriate clinical event. Regarding clinically relevant titers, anticardiolipin antibody IgG and/or IgM must be present in medium or high titer (>40 GPL or MPL, or >99th percentile) in serum or plasma. Anti-β2-glycoprotein I antibody IgG and/or IgM must be present in titer > 99th percentile in serum or plasma [31]. Notably, of the three antiphospholipid antibodies, lupus anticoagulant assays are impacted by anticoagulation in a unique way: warfarin, heparin, and DOACs can cause false positives or indeterminate results in these tests [28]. To verify unexpectedly low Protein C, Protein S, or antithrombin levels, the results should generally be confirmed after resolution of the acute event and cessation of anticoagulant therapy before a hereditary deficiency is diagnosed.

### 5.5. Clinical Implications

Inappropriate timing remains one of the most common causes of unreliable thrombophilia testing results. Multiple institutional audits have demonstrated that testing is frequently performed during hospitalization, acute thrombosis, or active anticoagulation, leading to uninterpretable results and unnecessary healthcare expenditures [3,32]. Accordingly, clinicians should first determine whether testing is likely to change management and then ensure that testing is performed under conditions that permit accurate interpretation.

Thus, thrombophilia testing should generally be deferred during acute VTE hospitalization unless the result is expected to change immediate management (e.g., suspected APS). For most patients, testing should occur after completion of initial anticoagulation and resolution of factors that may interfere with interpretation.

## 6. Evidence for Clinical and Therapeutic Yield

A growing body of evidence demonstrates a substantial disconnect between the frequency of thrombophilia testing, the detection of abnormal results, and the degree to which those findings meaningfully alter patient management. Although thrombophilia panels are frequently ordered following venous thromboembolism (VTE), multiple institutional audits, observational studies, and guideline reviews have consistently shown that testing is often performed inappropriately, interpreted incorrectly, or ordered in clinical settings where results are unlikely to influence therapeutic decisions [1,29].

### 6.1. Inappropriate Timing

One of the most consistent findings across literature is the high prevalence of inappropriate thrombophilia testing during acute hospitalization and active anticoagulation. In a large retrospective analysis at an academic medical center, Shen et al. demonstrated that thrombophilia testing was frequently ordered during periods in which results were expected to be unreliable, including acute thrombosis and concurrent anticoagulant therapy [3]. This study reviewed all thrombophilia testing records in a two-month span and included 173 patients. The authors found that a substantial proportion of testing lacked clear clinical indication and generated significant unnecessary cost, recording that testing was reduced by 84% after new guidelines were implemented. Similarly, in a narrative review examining previous literature, Petrilli et al. observed widespread inpatient inherited thrombophilia testing despite limited evidence that results would alter acute management [33]. These findings are particularly important because Protein C, Protein S, antithrombin, and lupus anticoagulant assays may all be significantly affected by acute thrombosis, inflammation, heparin exposure, vitamin K antagonists, and direct oral anticoagulants (DOACs), increasing the likelihood of false-positive or uninterpretable results [1,29].

### 6.2. Low Management Impact

Beyond inappropriate timing, multiple studies have demonstrated that thrombophilia testing rarely changes therapeutic decision-making. In a retrospective cohort study with 163 patients, Cox et al. evaluated thrombophilia testing practices at an academic medical center and found that testing seldom altered management despite frequent ordering [32]. This study did not assess whether the resulting clinical decisions were appropriate or collect any outcome data, but low management impact remains evidence of limited clinical utility. Similarly, guidance from the Anticoagulation Forum using existing guidelines emphasized that thrombophilia testing rarely alters management in patients with provoked VTE and should not be used routinely to determine anticoagulation duration [34]. Most patients with VTE do not require thrombophilia testing because results rarely influence duration of anticoagulation or recurrence risk assessment [1]. Collectively, these studies reinforce a central principle echoed through contemporary guidelines that thrombophilia testing should not be performed reflexively but rather reserved for situations in which results are expected to change management.

### 6.3. False Positive/Low-Value Findings

Importantly, diagnostic yield and therapeutic yield are not synonymous. Many thrombophilia panels identify abnormalities that have limited or uncertain clinical significance. Factor V Leiden and Prothrombin G20210A Variant generally confer only modest effects on recurrent VTE risk after a first thrombotic event, limiting their influence on decisions regarding extended anticoagulation [1,34]. Similarly, broad inherited thrombophilia panels frequently identify abnormalities that do not alter anticoagulation duration or intensity. MTHFR polymorphism testing represents an example of low-value testing. Despite persistent use in clinical practice, MTHFR variants have not demonstrated meaningful utility in predicting recurrent thrombosis or guiding management and are no longer recommended by major societies [1,34].

By contrast, antiphospholipid syndrome (APS) consistently demonstrates the highest therapeutic yield among thrombophilic disorders because diagnosis directly affects anticoagulant selection, treatment duration, pregnancy management, and arterial thrombosis prevention. Unlike most inherited thrombophilias, APS frequently changes clinical management in a meaningful way, particularly in patients with arterial thrombosis, recurrent thrombosis, pregnancy morbidity, or triple-positive antiphospholipid antibody profiles [1]. This distinction explains why APS testing occupies a uniquely prominent role across ASH, CHEST, NICE, and British Society for Haematology guidance statements.

### 6.4. Cost Implications

The financial burden associated with inappropriate thrombophilia testing has been highlighted in institutional utilization studies demonstrating substantial reductions in laboratory expenditures following implementation of evidence-based testing protocols [3]. In addition to direct laboratory expenditures, overtesting contributes to downstream costs including repeat testing, subspecialty referrals, prolonged anticoagulation, and patient anxiety. These concerns have led multiple professional organizations and Choosing Wisely initiatives to advocate strongly against routine thrombophilia testing following provoked VTE or in situations where management decisions have already been established [1,34]. Consequently, the central question is no longer whether a thrombophilic abnormality can be identified, but whether identification of that abnormality will alter a therapeutic decision.

## 7. Thrombophilia and Special Clinical Contexts

Several patient populations require individualized approaches to thrombophilia testing beyond the general framework described in the preceding sections. The subsections below address the four most frequently encountered in clinical practice: cryptogenic stroke in young adults, unusual-site thrombosis, pregnancy and reproductive planning, and cancer-associated VTE.

### 7.1. Young Adults with Ischemic Stroke

Ischemic stroke in young adults is increasing in global incidence, and up to half of cases in this age group remain cryptogenic after standard evaluation [35]. The absence of an identified mechanism is clinically important because it precludes targeted secondary prevention.

Antiphospholipid syndrome (APS) has been linked to cryptogenic stroke in young patients; proposed mechanisms include endothelial dysfunction, inhibition of the protein C pathway, and platelet activation [36]. Testing for antiphospholipid antibodies is therefore appropriate in young patients with cryptogenic stroke, with confirmatory repeat testing at 12 weeks required before a diagnosis of APS can be established.

The yield of extending the panel to inherited thrombophilias is substantially lower. Omran et al. evaluated 196 young adults with ischemic stroke who underwent thrombophilia screening at a comprehensive stroke center [37]. Although 43% had at least one positive test, when excluding isolated hyperhomocysteinemia, MTHFR heterozygosity and Factor V Leiden heterozygosity, all low risk thrombophilias, the positivity rate fell to 24%. Only 8% had a resultant change in management, and antiphospholipid antibodies accounted for most of those changes. No clinical predictor including cryptogenic stroke subtype, family history, or patent foramen ovale status was significantly associated with a positive inherited thrombophilia result or a management change. This was a clinician-selected subset and accounted for 12% of all stroke admissions during the study period, so the authors acknowledge the possibility of selection bias. However, the BSH similarly found that across 1900 stroke patients, testing changed management in only 0.2% of all tested patients [28]. Routine inherited thrombophilia screening in young adults with ischemic stroke is therefore not justified; the practical recommendation is to test selectively for antiphospholipid antibodies where cryptogenic stroke and a clear management question coexist.

### 7.2. Unusual-Site Thrombosis: Cerebral and Splanchnic Veins

Cerebral venous sinus thrombosis (CVST) and splanchnic vein thrombosis (portal, hepatic, splenic, or mesenteric) are uncommon presentations that frequently prompt broad thrombophilia evaluation. Both inherited thrombophilias, particularly Factor V Leiden and the Prothrombin G20210A Variant, and acquired conditions such as pregnancy, estrogen-containing contraceptives, and cirrhosis, contribute to these events [1].

Despite the high frequency with which thrombophilia testing is ordered in these settings, inherited thrombophilia screening rarely influences management. The BSH does not recommend testing for heritable thrombophilia when the only indication is the anatomical site of thrombosis, noting that the association is weak and that management is unlikely to change given that these patients are generally committed to extended anticoagulation [28]. The ASH 2023 guidelines conditionally recommend thrombophilia testing in CVST and splanchnic vein thrombosis only in the specific scenario where anticoagulation is being considered for discontinuation, since a high-risk positive result could then support indefinite treatment [25]. Antiphospholipid antibody testing is an important exception, as a positive result can alter both anticoagulant selection and treatment duration and should be pursued in the absence of clear provoking factors [28].

The initial investigations for CVST and splanchnic vein thrombosis should differ to some degree. In CVST, a hormonal trigger is the most commonly identified precipitant [36]. In splanchnic vein thrombosis specifically, the priority investigation is evaluation for acquired conditions such as myeloproliferative neoplasms (MPN) and paroxysmal nocturnal hemoglobinuria (PNH) rather than testing for inherited thrombophilias. Both MPNs and PNH are associated with this presentation and carry treatment implications beyond anticoagulation alone [1,28]. For patients with any unusual site thrombosis and evidence of hemolysis or abnormal hematological parameters, BSH suggests considering testing for PNH. For patients with any unusual site thrombosis and full blood count abnormalities concerning for MPN, BSH recommends testing with an MPN panel; if the full blood count is normal and there is no provoking factor, testing for *JAK2* mutation is suggested [28].

### 7.3. Pregnancy and Reproductive Planning

VTE During Pregnancy: In pregnant women who develop VTE, the acute management decision is not altered by thrombophilia status. Regardless of test results, VTE during pregnancy mandates therapeutic anticoagulation with low-molecular-weight heparin (LMWH), which does not cross the placenta, and thromboprophylaxis during subsequent pregnancies; combined oral contraceptives should be avoided [1].

Prior VTE with Future Pregnancy Planning: The CHEST 2012 antithrombotic therapy in pregnancy guidelines reinforce that prophylaxis decisions must be tied to specific management questions. They are stratified by thrombophilia type, personal history, and family history of VTE, not by testing performed reflexively [38]. According to the ASH 2018 guidelines for the management of VTE in pregnancy, antepartum prophylaxis is recommended for previous unprovoked or hormone-associated VTE; however, antepartum prophylaxis not recommended after a resolved nonhormonal transient event in the absence of additional risk factors [39].

Pregnancy Planning with Family History of VTE: For asymptomatic pregnant women with a family history of VTE, CHEST 2012 guidelines stratify thrombophilia testing and prophylaxis decisions dependent on the specific inherited thrombophilia and family history pattern. For pregnant women homozygous for factor V Leiden or the prothrombin 20210A mutation with a positive family history for VTE, antepartum prophylaxis with prophylactic- or intermediate-dose LMWH and postpartum prophylaxis for 6 weeks is suggested. For pregnant women with other thrombophilias and no prior VTE who have a positive family history for VTE, antepartum clinical vigilance and postpartum prophylaxis with prophylactic- or intermediate-dose LMWH or vitamin K antagonists (targeted at INR 2.0 to 3.0) is suggested. For pregnant women homozygous for factor V Leiden or the prothrombin 20210A mutation without a positive family history for VTE, antepartum clinical vigilance and postpartum prophylaxis for 6 weeks is suggested. For pregnant women with other thrombophilias and no prior VTE without a positive family history for VTE, antepartum and postpartum clinical vigilance is suggested rather than pharmacologic prophylaxis [38].

Recurrent Pregnancy Loss and Obstetric Antiphospholipid Syndrome: For women with recurrent pregnancy loss or severe obstetric morbidity, antiphospholipid antibody testing is appropriate, as an APS diagnosis directly changes both pharmacologic management and obstetric monitoring. Standard treatment for obstetric APS consists of low-dose aspirin combined with prophylactic-dose LMWH throughout pregnancy, yet pregnancy outcomes remain poor: even with conventional treatment, women who are triple-positive for antiphospholipid antibodies (lupus anticoagulant, anticardiolipin antibodies, and anti-β2-glycoprotein I antibodies) retain a high risk of adverse obstetric outcomes [40]. Because placental pathology in obstetric APS extends beyond frank thrombosis, additional immune-directed therapies are under active investigation [7]. Testing should generally be deferred until after pregnancy, as levels of antiphospholipid antibodies can fluctuate and render results unreliable [28].

### 7.4. Cancer-Associated VTE

Cancer, particularly mucin-producing adenocarcinomas, substantially elevates VTE risk through tumor-driven procoagulant mechanisms. Although inherited thrombophilia may add to this risk in relative terms, it does not influence management decisions in cancer-associated VTE. In these patients, anticoagulation duration is determined by the presence of active malignancy or ongoing cytotoxic therapy, not by thrombophilic genotype. The choice between direct oral anticoagulants and LMWH is based on bleeding risk and drug interaction profiles rather than thrombophilia status. Thus, since thrombophilia status does not play a role in anticoagulation strategy in these patients, there is no reason to test for inherited thrombophilia in patients with active cancer and VTE [1,41].

## 8. Targeted Testing Framework

The cumulative evidence supports a management-first approach to thrombophilia testing. Before ordering evaluation, clinicians should determine whether results are expected to alter anticoagulation duration, anticoagulant selection, reproductive counseling, or long-term risk management.

Three principles should guide appropriate testing:

1. Testing should only be ordered when the result is expected to change management.
2. Testing should be performed at the correct time to avoid unreliable results.
3. Low-value testing should be avoided.

APS remains the highest-yield thrombophilia diagnosis because identification frequently alters management. Testing is particularly appropriate in patients with cryptogenic stroke, unusual-site thrombosis, recurrent pregnancy morbidity, or hormonally associated thrombosis where duration of anticoagulation remains uncertain.

By contrast, routine thrombophilia testing after clearly provoked VTE generally provides little value and should be avoided. Broad thrombophilia panels performed during acute hospitalization or while patients are anticoagulated frequently generate uninterpretable or clinically insignificant results while increasing healthcare costs and patient anxiety.

## 9. Anticoagulation Overview

### 9.1. Anticoagulant Classes and Mechanisms

Three broad classes of anticoagulants are used in the prevention and treatment of VTE, each targeting a different point in the coagulation cascade.

Vitamin K antagonists (VKAs), most commonly warfarin, act in the liver by inhibiting the cyclic interconversion of vitamin K, which is required for the carboxylation and activation of clotting factors II, VII, IX, and X. Therapeutic monitoring via INR is required throughout treatment. VKAs are also considered teratogenic and are not the preferred treatment during pregnancy [39,42].

Heparins, including unfractionated heparin (UFH) and low-molecular-weight heparin (LMWH), work by binding and activating antithrombin, thereby accelerating its inhibition of thrombin (factor IIa) and factor Xa. UFH enhances both antithrombin-thrombin and antithrombin-factor Xa interactions and requires intravenous or subcutaneous administration with aPTT monitoring. LMWH preferentially inhibits factor Xa over thrombin, has more predictable pharmacokinetics, and can be administered subcutaneously without routine monitoring in most patients. LMWH does not cross the placenta, making it the preferred anticoagulant in pregnancy [42].

Direct oral anticoagulants (DOACs) act by directly inhibiting specific coagulation factors without requiring a cofactor. Factor Xa inhibitors including apixaban, rivaroxaban, and edoxaban bind and block factor Xa, interrupting the common pathway before thrombin generation. Direct thrombin inhibitors, most notably dabigatran, directly inhibit thrombin. Unlike VKAs, DOACs have a rapid onset of action, predictable pharmacokinetics, and require no routine coagulation monitoring, substantially simplifying outpatient management [42]. However, like VKAs, DOACs are not preferred in pregnancy due to concerns about likely placental transfer as well as a lack of evidence regarding their safety and reproductive effects. For breastfeeding women, the ASH guideline panel recommends against the use of DOACs [39]. Because DOACs directly affect functional coagulation assays, they can interfere with thrombophilia testing for Protein C, Protein S, antithrombin, and lupus anticoagulant, an important practical consideration.

### 9.2. The DOAC Era: Its Efficacy, Safety, and Role in Thrombophilia

The emergence of direct oral anticoagulants (DOACs) has substantially reshaped thrombosis management and indirectly reduced the clinical utility of many inherited thrombophilia diagnoses. Anticoagulation decisions are increasingly determined by recurrence risk and provoking factors rather than thrombophilia subtype alone.

CHEST 2021 recommends DOACs as first-line therapy for most patients with VTE because of comparable efficacy, improved safety profiles, and greater convenience compared with vitamin K antagonists [26]. A meta-analysis of seven randomized controlled trials compared factor Xa inhibitors to traditional anticoagulants for preventing VTE recurrence. For the factor Xa inhibitors, there was a statistically significant 15% reduction in recurrence risk, pooled hazard ratio (HR) 0.85, 95% CI 0.72–0.99, *p* = 0.04. A subgroup analysis of non-cancer patients with VTE demonstrated a non-significant 10% relative risk reduction in VTE recurrence for factor Xa inhibitors compared to traditional therapies, pooled HR 0.90, 95% CI 0.76–1.06, *p* = 0.19. However, analysis of cancer-associated VTE demonstrated a statistically significant 24% reduction in VTE recurrence risk favoring factor Xa inhibitors, pooled HR 0.76, 95% CI 0.59–0.98, *p* = 0.03 [43].

APS remains the major exception even in this modern era. Evidence increasingly supports warfarin over DOAC therapy in high-risk APS, particularly in triple-positive patients and those with arterial thrombosis [7,44]. This distinction represents an example of thrombophilia status directly altering anticoagulant selection and duration of therapy.

## 10. Treatment Duration Framework: Provoked, Unprovoked, and Hormonal Triggers

The single most consequential clinical decision following a VTE event is whether anticoagulation should be continued indefinitely or stopped after a fixed primary treatment period. This decision is governed by an assessment of the patient’s long-term recurrence risk relative to their bleeding risk. The evidence reviewed throughout this paper makes it clear that it is determined primarily by the nature of the provoking event, not by thrombophilic genotype. Understanding the provocation-based framework that underpins contemporary treatment duration decisions is therefore essential context for evaluating where thrombophilia testing does and does not belong.

### 10.1. The Three-Phase Treatment Model

The CHEST 2021 guideline structures VTE anticoagulation across three sequential phases [26]. The initiation phase covers approximately the first 5–21 days of treatment and is concerned with acute clot stabilization and prevention of propagation. The primary treatment phase, which extends to three months, addresses active secondary prevention during the period of highest residual thrombotic risk. These two phases apply to virtually all patients with DVT or PE who do not have a contraindication to anticoagulation. The extended phase constitutes secondary prevention with no planned stop date for the anticoagulation, where provocation status, thrombophilia results, bleeding risk, and patient preferences collectively determine management. All patients completing the first three months should be formally reassessed for extended-phase anticoagulation at that point.

### 10.2. Provoked VTE: Major and Minor Transient Risk Factors

The International Society on Thrombosis and Hemostasis (ISTH) scientific and standardization committee guidance formally defined the categorization of VTE as provoked or unprovoked and established the framework now adopted across all major guidelines [45]. VTE is considered provoked by a major transient risk factor when it occurs in temporal association with surgery under general anesthesia for more than 30 min, hospitalization with strict bed rest for at least three days for an acute medical illness, major trauma, or cesarean section. VTE provoked by a minor transient risk factor includes events associated with minor surgery, brief immobilization, long-distance travel, or acute medical illness not requiring bed rest.

For VTE provoked by a major transient surgical risk factor, recurrence risk after stopping anticoagulation is low (approximately 1% per year) and extended-phase anticoagulation is not recommended [26,46]. The reversal of the provoking factor is sufficient justification for stopping treatment. For VTE provoked by a major (acute medical illness, hospitalization) or minor (estrogen-containing therapy, pregnancy, puerperium) non-surgical transient risk factor, the recurrence risk after stopping anticoagulation is meaningfully higher, estimated at approximately 5% in the first year although true recurrence risk varies based on the specific risk factor [46]. Whether extended anticoagulation is appropriate in this setting depends on the persistence of the underlying risk factor, the patient’s bleeding risk, and whether a thrombophilia testing result might tip the balance. In these intermediate-risk scenarios, the ASH 2023 guidelines conditionally recommend thrombophilia testing precisely because results may alter the decision to continue or stop treatment [25]. In all other clearly provoked settings including index events provoked by minor transient risk factors where the provoking exposure has resolved, extended anticoagulation is not recommended, and thrombophilia testing is not expected to change that decision [26].

### 10.3. Unprovoked VTE: The Case for Extended Anticoagulation

VTE is classified as unprovoked when no transient or persistent provoking risk factor can be identified at the time of the event. This classification carries important prognostic weight. A landmark systematic review and meta-analysis drawing on data from more than 7000 patients (with a first unprovoked VTE who discontinued anticoagulation after initial treatment) reported a cumulative recurrence rate of approximately 10% in the first year, 16% at two years, 25% at five years, and 36% at ten years [47]. These figures are substantially higher than the corresponding rates for surgically provoked VTE. This forms the principal evidence base for recommending extended anticoagulation in patients with unprovoked events who are at low or moderate bleeding risk [26].

The implication for thrombophilia testing in this setting is important and frequently misunderstood. The CHEST 2021 framework already favors extended anticoagulation for most patients with unprovoked VTE, which means that a positive inherited thrombophilia result does not change that recommendation, and a negative result should not be used to justify stopping treatment. The ASH 2023 guidelines quantified this harm through decision modeling. Discontinuing anticoagulation based on a negative thrombophilia result was estimated to produce approximately 42 additional recurrences of VTE events per 1000 patients per year, compared to a strategy of indefinite anticoagulation for all unprovoked VTE. This harm substantially outweighs the reduction in major bleeding achieved by stopping anticoagulation therapy [25]. For this reason, ASH 2023 conditionally recommends against thrombophilia testing following unprovoked VTE, and all major guideline frameworks converge on the same conclusion that a negative result should not be used to reassure clinicians or patients that anticoagulation can safely be stopped.

### 10.4. Hormonal and Nonsurgical Provoked VTE: The Testing Window

VTE associated with hormonal exposures such as use of combined oral contraceptive (COC), pregnancy, the postpartum period, or hormone replacement therapy constitute a clinically distinct position in the treatment duration framework. These events are technically provoked, but the provoking factor may recur. For example, a woman who experienced VTE on COCs may wish to resume hormonal contraception or become pregnant, and a woman with pregnancy-associated VTE may face future pregnancies. Whether to use extended anticoagulation, and which contraceptive or reproductive strategy to adopt, may therefore be genuinely altered by thrombophilia status in ways that are not true of surgically provoked VTE.

The ASH 2023 guidelines conditionally recommend thrombophilia testing in patients with VTE provoked by nonsurgical major transient risk factors and in patients with hormonally associated VTE after completion of primary treatment [25]. This recommendation reflects the finding that the recurrence risk in hormonal and nonsurgical provoked VTE is intermediate, which is too high to recommend stopping anticoagulation reflexively, but not high enough to recommend indefinite treatment in all cases without individualization. In a woman with COC-associated VTE who wishes to resume hormonal therapy, certain high-risk results would change management significantly. Triple-positive antiphospholipid antibody status, antithrombin deficiency, or homozygous Factor V Leiden would each support indefinite anticoagulation. They would also support permanent avoidance of combined hormonal contraception. In contrast, a negative panel would support treatment cessation after the primary phase and, the patient could consider revisiting hormonal contraceptives but while receiving extensive counseling based on history, estrogen dose and route, available non-estrogen alternatives, and plans for pregnancy. This represents a clear example in the thrombophilia testing literature of a situation in which the result of testing is expected to produce a specific, actionable change in management. Importantly, these statements are reflective of the ASH 2023 modeling strategy and are based on very low certainty evidence; they are not conclusions drawn from direct outcome studies.

### 10.5. The Role of Bleeding Risk Assessment

The provocation-based framework above should generally be interpreted alongside bleeding risk. Before placing any patient on indefinite anticoagulation, bleeding risk must be formally evaluated. The VTE-BLEED score was developed specifically for this purpose in VTE patients, using variables such as active cancer, age, sex, presence of anemia, renal impairment, prior bleeding history, and blood pressure control to stratify bleeding risk in patients receiving long-term anticoagulation [48]. The HAS-BLED score is another widely used tool that incorporates similar clinical variables; although originally developed in patients with atrial fibrillation, it has been broadly applied in VTE populations in clinical practice [49]. For both tools, elevated scores demonstrate increased bleeding risk with anticoagulation. However, the use of indefinite anticoagulation is not dependent on these scores alone, and individualized bleeding-risk assessment should be completed for every patient. The CHEST 2021 guidelines also provide a structured clinician-directed approach to support this assessment [26].

Even in patients with unprovoked VTE at high recurrence risk, the absolute benefit of extended anticoagulation diminishes when annual major bleeding risk exceeds approximately 3%. A positive thrombophilia result does not override a high bleeding risk from long-term anticoagulation, as it may alter the risk-benefit equation rather than resolving it. Shared decision-making, incorporating patient values and the practical burdens of long-term anticoagulation, remains central to the final treatment decision.

In summary, the framework that governs treatment duration in VTE is built on three factors: provocation status, recurrence risk, and bleeding risk. Thrombophilia genotype does not drive this framework. Testing has a role in only one specific situation when determining duration of treatment with anticoagulation, which is when VTE is provoked by hormonal or nonsurgical factors. In this intermediate-risk group, the decision to continue or stop anticoagulation is genuinely uncertain, and a thrombophilia result may influence that decision. In all other situations, including clearly surgically provoked VTE and most cases of unprovoked VTE where extended treatment is already indicated, thrombophilia testing will not change the treatment duration decision and should generally not be ordered for that purpose (Figure 2 and Table 4).

## 11. Research Gaps and Future Directions

Despite the evidence synthesized in this review, several important knowledge gaps remain that future research should address. First, prospective studies are needed to validate the characterization of intermediate-risk patients such as those with hormonal or nonsurgical provoked VTE where anticoagulation duration decisions remain uncertain. Current recommendations for this group rely largely on modeling rather than direct outcome data; prospective cohort studies comparing management strategies in these patients would further strengthen evidence-based guidelines. Secondly, research comparing anticoagulant selection across different APS subgroups could greatly strengthen the current understanding of decision making within this patient population. Currently, Warfarin is favored over DOACs in high-risk APS, but this recommendation is based on limited data and would benefit from more rigorous comparative studies. Another potential area of research could focus more on implementation of current guidelines, specifically the examination of barriers to adherence with guideline-recommended selective testing approaches. This could further help understanding why testing persists despite guideline recommendations and may reveal opportunities for system-level improvement.

Finally, emerging evidence is reshaping post-VTE risk stratification beyond conventional inherited thrombophilia testing. Recent clinical prediction models incorporating clinical and biochemical variables, novel biomarkers (D-dimer, P-selectin, thrombin generation assays), and machine learning algorithms show promise for individualized recurrence-risk assessment. These tools may better identify patients who can safely discontinue anticoagulation or who require extended therapy compared to traditional binary provoked/unprovoked classification. Similarly, artificial intelligence-supported decision-support systems are being investigated to optimize thrombophilia testing utilization and improve guideline adherence at the point of care [50]. Integration of these emerging technologies with the management-oriented framework proposed in this review could enhance personalization of post-VTE care. Future research should evaluate the clinical utility, cost-effectiveness, and implementation feasibility of these evolving risk stratification approaches in real-world practice settings.

## 12. Conclusions

Thrombophilia testing after VTE is not a routine diagnostic obligation. It is a selective clinical tool whose value depends entirely on whether the result is expected to change management and when appropriately timed and targeted can provide meaningful guidance. The evidence reviewed in this paper is consistent across multiple institutional audits and real-world outcome studies and is supported by major contemporary guidelines. Testing that does not answer a specific therapeutic question generates cost, false reassurance, and potential harm without improving patient outcomes. However, appropriately indicated testing can inform anticoagulation decisions and patient management. The suitability of testing is influenced by appropriate timing, status of anticoagulation, choice of test, assay method, clinical urgency, interpretability of results, and management impact. The highest-yield indication remains antiphospholipid syndrome testing, where a confirmed diagnosis directly affects anticoagulant selection, treatment duration, and pregnancy management. Inherited thrombophilia testing should be reserved for carefully selected patients and performed at the right time. Functional testing should generally be deferred or interpreted with specialist laboratory input while anticoagulation is active (certain solid-phase antibody and genetic tests may be performed when clinically necessary). MTHFR polymorphism testing has no demonstrated clinical utility in VTE and should be avoided.

Ultimately, the goal is not to discourage thrombophilia testing but to restore its clinical purpose. Before ordering any test, the clinician should be able to answer one question: will this result change what to do next? When the answer is yes, testing is appropriate. When it is not, testing is not recommended to proceed. The major guidelines, while differing in a few specific scenarios, consistently emphasize this simple principle, empowering clinicians to improve the quality of thrombophilia care while preserving value for patients.

## Figures and Tables

**Figure 1:**
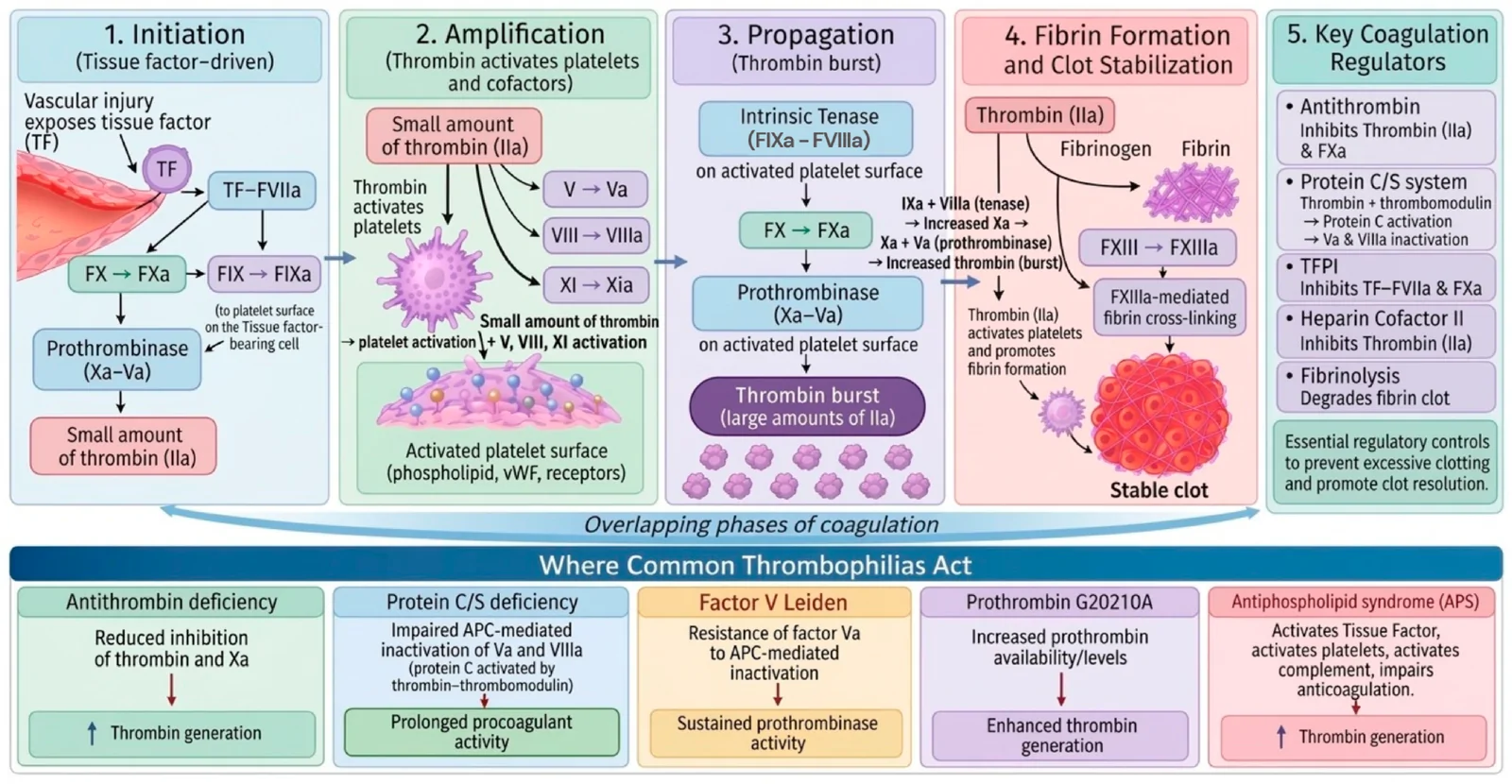
Cell-based Model of Hemostasis and Mechanisms of Common Thrombophilias.

**Figure 2:**
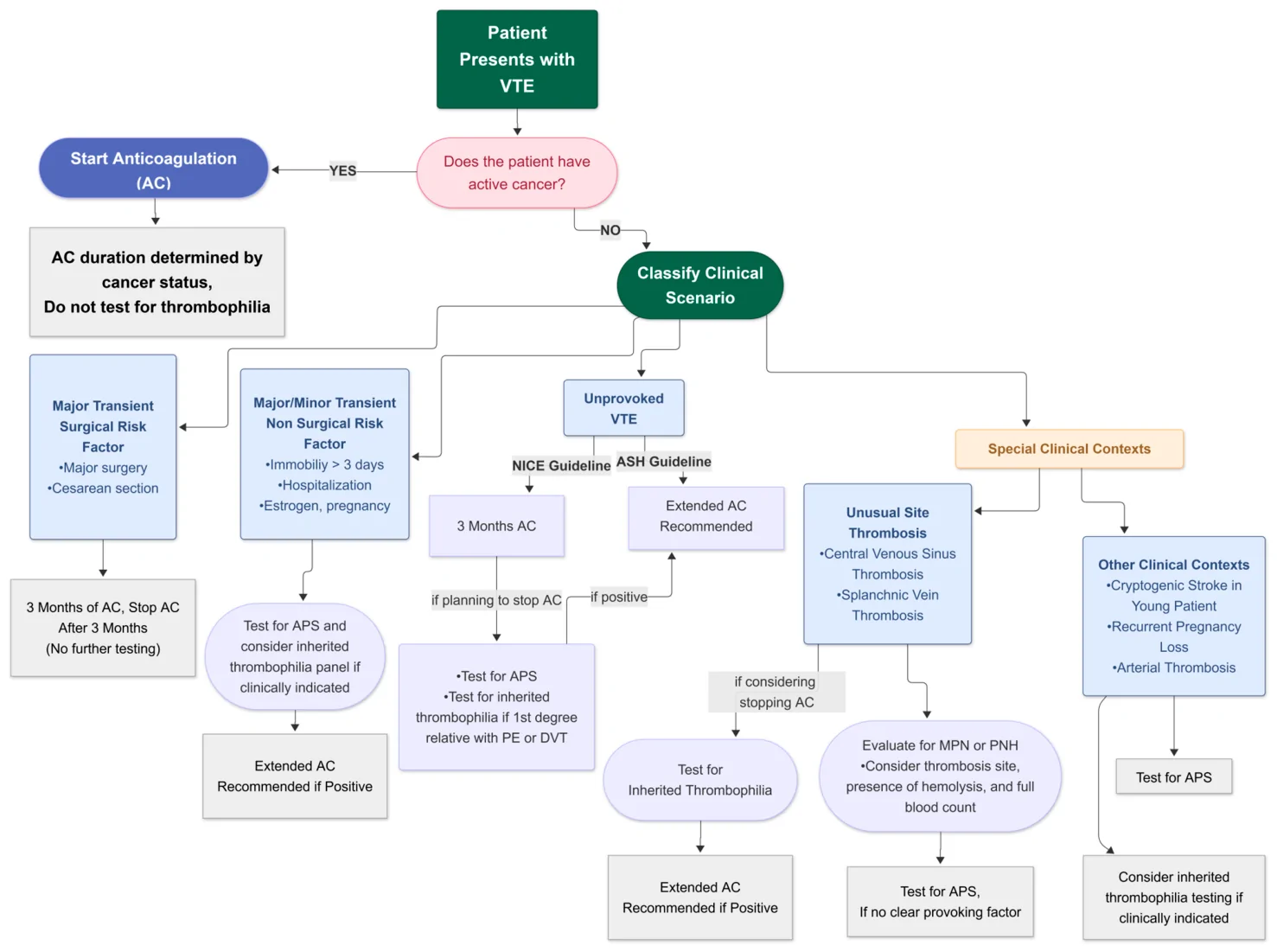
Suggested algorithm for Venous Thromboembolism (VTE) Workup and Management. Legend: AC = anticoagulation; APS = antiphospholipid syndrome; MPN = myeloproliferative neoplasm; PNH = paroxysmal nocturnal hemoglobinuria.

**Table 1:** Summary of common thrombophilias: general population prevalence, annual VTE risk in asymptomatic carriers, relative risk versus non-carriers, and clinical risk classification.

Thrombophilia	General Population Prevalence	Annual Incidence (Asymptomatic Carriers)	Risk vs. Non-Carriers	Risk Category
**Factor V Leiden—heterozygous**	3–8% (European-descent); 0.5–2% (non-European)	0.28% total/0.11% spontaneous	RR 2.8 (95% CI 1.1–8.6)	Low–Moderate
**Factor V Leiden—homozygous**	Rare	1.8%/year [95% CI 0.1–4.0]	OR 7.38 (95% CI 4.68–11.65)	High
**Prothrombin G20210A** **(heterozygous)**	1–3% (European-descent)	~0.4%/year [95% CI 0.1–1.1]	3×	Low–Moderate
**Antithrombin deficiency**	0.02–0.04%	1.6%/year	10.6× (combined AT/PC/PS) [95% CI 4.0–23.6]	High
**Protein C deficiency**	0.2–0.4%	~1.0%/year	10.6× (combined AT/PC/PS) [95% CI 4.0–23.6]	High
**Protein S deficiency**	0.03–0.13%	~0.4%/year	10.6× (combined AT/PC/PS) [95% CI 4.0–23.6]	High
**Antiphospholipid syndrome (APS)**	~1–5% (aPL seroprevalence); APS ~0.05% general population	Variable; high-risk (triple positive) > 29%/year thrombotic recurrence	Data unavailable	High

Legend: aPL = antiphospholipid antibodies; APS = antiphospholipid syndrome; FVL = Factor V Leiden; MTHFR = methylenetetrahydrofolate reductase; VTE = venous thromboembolism.

**Table 2:** Comparative guideline for thrombophilia testing.

Clinical Scenario	ASH 2023	CHEST 2021	NICE NG158	BSH 2022
**Unprovoked VTE**	Against routine testing for both inherited and acquired thrombophilia	Extended anticoagulation favored with DOAC	Test for APS/hereditary thrombophilia if stopping anticoagulation	Test for APS
**Major Provocation VTE**	Against testing for both inherited and acquired thrombophilia	No extended anticoagulation	Against thrombophilia testing (does not specify acquired vs. inherited)	Guideline does not specify for major provocation
**Minor Provocation VTE**	Consider testing for both inherited and acquired thrombophilia to determine anticoagulation duration	Treat with short term anticoagulation	Guideline does not specify for minor provocation	Test for APS

**Table 3:** Optimum time to test for thrombophilia screening.

Test	Recommended Timing
**Factor V Leiden Variant**	Any time, although usually deferred until outpatient evaluation
**Prothrombin G20210A Variant**	Any time, although usually deferred until outpatient evaluation
**Protein C activity**	Generally after 3 months of anticoagulation and after the acute thrombotic phase; testing should account for anticoagulant-related interference by allowing a wash out period of 2 weeks from warfarin or 2–3 days from DOACs.
**Free Protein S**	Generally after 3 months of anticoagulation and after the acute thrombotic phase; testing should account for anticoagulant-related interference by allowing a wash out period of 2 weeks from warfarin or 2–3 days from DOACs.
**Antithrombin** **activity**	Generally after 3 months of anticoagulation and after the acute thrombotic phase; testing should account for anticoagulant-related interference by allowing a wash out period of 2 weeks from warfarin or 2–3 days from DOACs.
**Lupus** **anticoagulant**	Off interfering anticoagulants whenever possible
**Anticardiolipin** **antibodies**	Any time; positive results require repeat testing after ≥12 weeks
**Anti-β2 glycoprotein I antibodies**	Any time; positive results require repeat testing after ≥12 weeks

**Table 4:** Anticoagulation Recommendations According to Clinical Scenario and Thrombophilia Evaluation.

Clinical Scenario	Recommended Duration of Anticoagulation	Preferred Anticoagulant	Evidence Level/Recommendation Strength
**Active cancer-associated VTE**	Continue while cancer is active or patient is receiving cancer therapy	DOAC (apixaban, rivaroxaban, edoxaban) is preferred in most patients; many variables such as tumor location, hepatic and renal function, drug interactions, patient preference, and other factors should be considered when determining preferred treatment	Conditional; low to very low certainty
**Major transient surgical provoking factor (major surgery, cesarean section)**	3 months	DOAC preferred (LMWH if pregnant or breastfeeding)	Strong; moderate certainty
**Major transient nonsurgical provoking factor (major trauma, prolonged immobilization, acute medical illness, hospitalization)**	Usually 3 months; extended therapy only in selected patients	DOAC preferred	Strong against extended therapy; moderate certainty
**Estrogen-associated VTE**	Minimum 3 months; extended therapy individualized according to recurrence risk	DOAC preferred unless APS present	Weak against routine extended therapy; moderate certainty
**Unprovoked VTE**	Extended or indefinite anticoagulation for most patients with low bleeding risk	DOAC preferred	Strong; moderate certainty
**Recurrent unprovoked VTE**	Indefinite anticoagulation	DOAC preferred unless APS present	Strong; moderate certainty
**Antiphospholipid syndrome with VTE**	Indefinite anticoagulation in most patients	Warfarin (INR 2.0–3.0) preferred; avoid DOACs in high-risk APS	Weak; low certainty for VKA over DOAC
**Triple positive APS**	Indefinite anticoagulation	Warfarin preferred; DOACs generally discouraged	Weak; low certainty
**Cerebral venous sinus thrombosis (CVST)**	Typically 3–12 months; indefinite therapy in selected patients	DOAC or warfarin depending on clinical circumstances	No formal graded thrombophilia-specific recommendation; evidence limited
**Splanchnic vein thrombosis**	Usually at least 6 months; longer if persistent risk factor present	DOAC, LMWH, or warfarin depending on etiology	No formal graded thrombophilia-specific recommendation; evidence limited
**Cryptogenic ischemic stroke**	Determined by stroke mechanism	Warfarin generally preferred if APS confirmed	No formal graded thrombophilia-specific recommendation; evidence limited
**Arterial thrombosis with APS suspicion**	Usually indefinite if APS confirmed	Warfarin preferred	Weak; low certainty
**Recurrent pregnancy loss with APS**	Obstetric anticoagulation strategy rather than VTE treatment duration	Low-dose aspirin plus prophylactic LMWH during pregnancy	Conditional; low-certainty evidence
**Isolated heterozygous Factor V Leiden or Prothrombin G20210A Variant after first provoked VTE**	Same as non-thrombophilia patients (typically 3 months)	DOAC preferred	Strong; moderate certainty for standard treatment duration; thrombophilia does not alter duration
**Protein C, Protein S, or Antithrombin deficiency**	Determined primarily by recurrence risk rather than thrombophilia status alone	DOAC generally preferred; evidence remains limited and management should be individualized according to the clinical event, recurrence, drug interactions, renal function, and specialist recommendations	No thrombophilia-specific graded duration recommendation; evidence limited

Legend: APS = antiphospholipid syndrome; CVST = cerebral venous sinus thrombosis; MPN = myeloproliferative neoplasm; PNH = paroxysmal nocturnal hemoglobinuria.

## Data Availability

No new data were created or analyzed in this study. Data sharing is not applicable to this article.

## Abbreviations

VTE	Venous Thromboembolism
APS	Antiphospholipid Syndrome
FVL	Factor V Leiden
DOAC	Direct Oral Anticoagulant
LMWH	Low-Molecular-Weight Heparin
CVST	Cerebral Venous Sinus Thrombosis
MPN	Myeloproliferative Neoplasm
PNH	Paroxysmal Nocturnal Hemoglobinuria
MTHFR	Methylenetetrahydrofolate reductase
